# Long non-coding RNAs as the regulatory hubs in rice response to salt stress

**DOI:** 10.1038/s41598-022-26133-x

**Published:** 2022-12-15

**Authors:** Raheleh Mirdar Mansuri, Amir-Hossein Azizi, Amir-Hossein Sadri, Zahra-Sadat Shobbar

**Affiliations:** Department of Systems Biology, Agricultural Research, Education and Extension Organization (AREEO), Agricultural Biotechnology Research Institute of Iran (ABRII), PO Box 31535-1897, Karaj, Iran

**Keywords:** Bioinformatics, Gene expression analysis, Plant genetics, Plant stress responses, Long non-coding RNAs

## Abstract

Salinity seriously constrains growth and fertility of rice worldwide. Long non-coding RNAs (lncRNAs) play crucial roles in plant abiotic stress response. However, salt responsive lncRNAs are poorly understood in rice. Herein, salt responsive lncRNAs (DE-lncRNAs) were identified in FL478 (salt tolerant) compared to its susceptible parent (IR29) using RNA-seq in root tissues at seedling stage. In FL478 and IR29, 8724 and 9235 transcripts with length of > 200 bp were nominated as potential lncRNAs, respectively. Rigorous filtering left four (in FL478) and nine (in IR29) DE-lncRNAs with only 2 DE-lncRNAs in common. ATAC-seq data showed that the genomic regions of all four lncRNAs in FL478 and 6/9 in IR29 are significantly accessible for transcription. Weighted correlation network analysis (WGCNA) revealed that lncRNA.2-FL was highly correlated with 173 mRNAs as *trans*-targets and a gene encoding pentatricopeptide repeat (PPR) protein was predicted as *cis*-target of lncRNA.2-FL. In silico mutagenesis analysis proposed the same transcription factor binding sites (TFBSs) in vicinity of the *trans*- and *cis*-regulatory target genes of lncRNA.2-FL, which significantly affect their transcription start site (TSS). This study provides new insights into involvement of the DE-lncRNAs in rice response to salt stress. Among them, lncRNA.2-FL may play a significant regulatory role in the salt stress tolerance of FL478.

## Introduction

The rising world's population is driving up global food demand, which is expected to approximately double by the year 2050^1^. Global food supply will require a fundamental conception of climatic factors influencing agricultural production^2^. Salinity is one of the major constraints in 33% of the world’s arable lands^3^. Rice (*Oryza sativa*) is a crucial crop that provides a main calorie source for billions of people^4^. Rice is generally sensitive to salt stress, with an electrical conductivity (EC) threshold of 3 dSm^− 1^ for most cultivated varieties^3^. Impacts of salinity stress on rice is affected by its growth and development stages. Overall, seedling stage is the most sensitive stage to salt stress in rice^5^. Excessive salinity leads to a significant decrease in main yield components in rice. Therefore, enhancing salt tolerance in rice is necessary to achieve food security for billions of people around the world.

Numerous studies have been performed to understand the molecular mechanisms of salt stress response in plants; however, they have chiefly focused on the functional analysis of protein-coding genes^6–8^. Over the last years, several studies have shown that non-coding RNAs (ncRNAs) act as regulatory molecules responding to abiotic or biotic stresses in plants^9,10^. Researchers have identified a high number of ncRNAs involved in salt stress response^11^. NcRNAs are functional RNA molecules transcribed from DNA but not translated into proteins. NcRNAs comprise housekeeping, regulatory, and functionally unknown ncRNAs. Regulatory ncRNAs are usually grouped based on their lengths into small RNAs (sRNAs) and long non-coding RNAs (lncRNAs)^12^. lncRNAs are often defined as transcripts longer than 200 nucleotides in length that do not encode proteins^13^. LncRNAs can perform their functions in *trans*-acting or *cis*-acting in the plant genome using varied mechanisms, such as sequence complementarity or similarity with DNAs or RNAs, promoter activity modification by nucleosome repositioning, and epigenetic regulation through DNA methylation and histone modification^14^. Numerous stress responsive lncRNAs have been identified in many plants (e.g., Medicago, *Zea mays,* and Arabidopsis)^15–17^. A novel drought-induced lncRNA has been found in Arabidopsis, which plays a major role under drought or salt stress^18^. Moreover, 3714 lncRNAs have been reported in rice under drought or salt stress, among which 1010 lncRNAs were differentially expressed in at least one stress condition/variety^19^. Although a number of lncRNAs associated with response to drought, salt, and osmotic stress in rice have been identified, the precise role of these lncRNAs is still not fully described or understood^6,20^.

Deep neural networks (DNNs) are famously good in extracting novel and hidden features from long sequential data. Recently, a DNN has been developed that can accurately predict the expression levels of candidate genes from the neighboring genomic sequence^21^. This network can also predict the change in the expression profile of different genes as a result of introducing mutations.

We have previously investigated how FL478 responds to the salinity through protein-coding genes, compared to its susceptible parent (IR29)^22^. In this study, we tried to find out the roles of salt stress responsive lncRNAs in these contrasting genotypes. Herein, differentially expressed lncRNAs (DE-lncRNAs) were identified in FL478 compared to IR29. The DE-lncRNAs were subjected to co-expression network analysis, once with their neighboring protein coding genes (*cis*-regulation) and once with all the differentially expressed transcripts (DETs) (*trans*-regulation) to identify potential functions of salt-responsive lncRNAs. Further, we used a recently developed deep neural network (DNN), Enformer, to predict the functional importance of genomic regions on the expression level of genes. The crosstalk between DE-lncRNAs and miRNAs was also investigated via exploring the DE-lncRNAs acting as the target pattern of known miRNAs in *Oryza sativa*. The achieved results could expand the knowledge about lncRNAs participating in mechanisms underlying salt tolerance in FL478.

## Materials and methods

### Plant growth, salt stress treatment, and RNA extraction

Seeds of FL478 and IR29 as salt-tolerant and salt-sensitive rice genotypes, respectively, were provided from International Rice Research Institute (IRRI). Sterilization and germination of the seeds and plant growth conditions were carried out, as formerly described^22^. The experiment was done in a factorial arrangement relying on a complete randomized block design with three biological replicates containing 10 plant samples for each genotype. Seedling stage was selected for the transcriptome analysis as it is the most sensitive stage of rice to salt stress^5,23^. The young seedlings of FL478 and IR29 were grown in a growth chamber (14 h light/10 h dark at temperature 28 ± 2 °C) for 3 weeks. The early response of rice to the moderate/high level of salt stress were aimed to study (while it could be endured by the sensitive genotype), so, 21-day-old rice seedlings were transferred to Yoshida solution with or without 150 mM NaCl^24,25^ for 24 h (for RNA extraction) or 1 week (for phenotypic observation, Supplementary Fig. S1). Total root samples of the seedlings were collected 24 h after incepting salt stress, instantly put in liquid nitrogen, and kept at − 80 °C until RNA extraction. Total RNA extraction was performed using the RNeasy Plant kit (Qiagen) from 100 mg of the root tissues.

### Sequencing and lncRNA identification

The RNA integrity and quality were examined, and two biological replicates of control and salt treated IR29 and FL478 root samples with RNA integrity number (RIN) > 7.6 were used for RNA sequencing based on the IlluminaHiSeq™ 2500 sequencing platform at Novogene Bioinformatics Institute (Beijing, China). The raw sequencing reads in FASTQ format were initially checked by FASTQC utility. The high-quality reads were mapped to the rice reference genome IRGSP 1.0 10 (ftp://ftp.ensemblgenomes.org/pub/plants) using TopHat^26^. Bowtie was used to index the genome based on the *O. sativa* cv. Nipponbare (ssp. japonica) reference genome annotation (http://plants.ensembl.org/Oryza_sativa/Info/Annotation)^27^. Cufflinks utility was utilized to assemble the reads. Cuffcompare was used to identify novel transcripts, and the assembled transcripts were selected for downstream analysis in the two samples. Expression levels of the transcripts were calculated according to fragments per kilobase per million mapped reads (FPKM). Only transcripts occurring in at least two samples were retained as expressed transcripts (for multiple-exon transcripts FPKM ≥ 0.5, for single exon transcripts FPKM ≥ 2).

**Figure 1:**
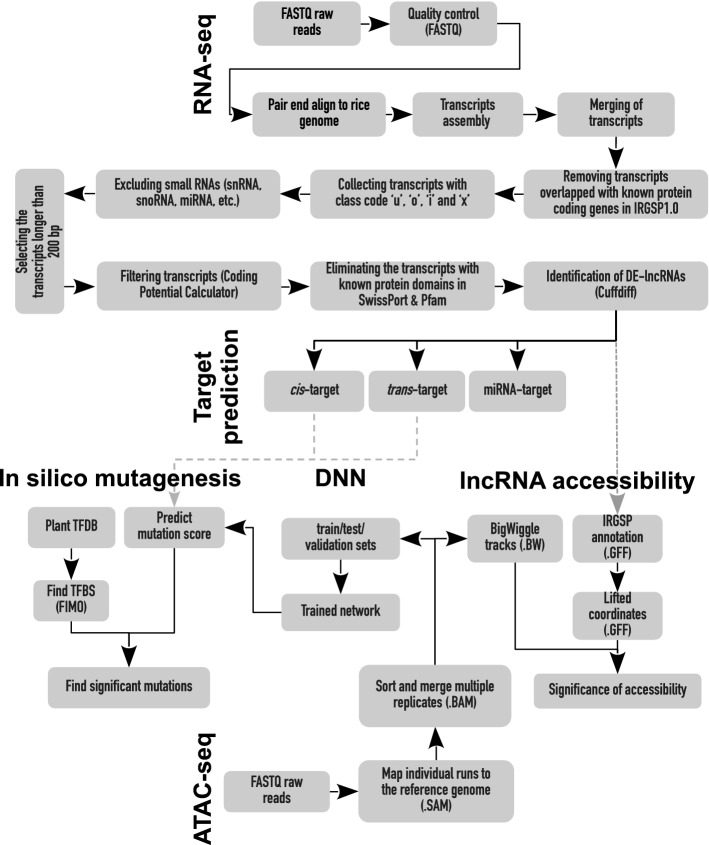
The integrated analysis pipeline for identifying the salt-responsive lncRNA in rice.

The following steps were used to identify lncRNAs based on their characteristics, as described by Kang and Liu in 2019^28^(Fig. 1): (1) Overlapped transcripts with known protein-encoding genes on IRGSP1.0 were identified and removed (2); Transcripts with a class code of “i” (located entirely within the intronic regions), “x” (natural antisense transcripts (NAT)), “u” (intergenic transcript), or “o” (generic exonic overlap with a reference transcript) were selected; (3) Small RNAs (snRNAs, snoRNAs, and miRNAs, etc.) were detected and filtered as the assembly template using rice annotation (IRGSP-1.0; http://plants.ensembl.org) and sequence alignment against the Rfam 14.3 database using the Blast2GO program^29^; (4) Transcripts with the length > 200 bp were selected; (5) The coding potential calculator (CPC)^30^ was then used to identify transcripts with low coding potential scores; (6) Transcripts with known protein domains based on Pfam-hidden Markov model (HMMs) databases (Eddy 2009) and SwissProt NR database^31^ were excluded; (7) Intersection of the transcripts filtered by CPC, Pfam, and SwissProt were assigned as potential lncRNAs. Various kinds of lncRNAs (i.e., lincRNAs (Long intergenic non-coding RNA), intronic lncRNAs, anti-sense lncRNAs, and sense lncRNAs) were nominated by cuffcompare (Fig. 1).

### Identification of differentially expressed lncRNAs

Differentially expressed lncRNAs were pair-wisely identified by Cuffdiff 2.1.1 software to estimate fragments per kilobase of exon per million fragments mapped (FPKMs) in the rice roots of the two genotypes^28^. The Q-value cut-off ≤ 0.05 and log2 fold change ≥ 1 (up-regulated genes) and ≤ (− 1) (down-regulated genes) were set as the threshold for significantly differential expression.

### Target genes prediction of salt-responsive DE-lncRNAs

The transcripts of neighboring protein-coding genes located in the 100 kb upstream and downstream of DE-lncRNAs were saved and subjected to co-expression analysis, to identify the putative target genes of DE-lncRNAs in *cis*-regulation. Correlation analysis was carried out using R language (reshape2 package) to identify the functional connection between DE-lncRNAs and the transcription of neighboring protein-coding genes^28^. Moreover, DE-lncRNAs together with the identified expressed transcripts (ETs) through the RNA-seq were subjected to the weighted correlation network analysis (WGCNA) standard process to detect potential target genes in *trans*-regulation (Langfelder and Horvath 2008). They were also clustered to search for common expression modules, and their function was analyzed through GO functional enrichment analysis. GO enrichment analysis was implemented using the AgriGO public web tool^32^. Further, genes located in common modules were functionally classified into categories using KEGG pathways.

### ATAC-seq data analysis

To check for the accessibility of the intended genomic regions and to train the deep neural network, ATAC-seq reads from six tissues (root, young leaf, flag leave, young panicle, lemma and palea, and stamen and pistil) collected by Zhao et al.^33^, were used. The reads were mapped to the Zhenshan 97 (ZS97) genome^34^ using BWA-MEM^35^. Reads with mapping quality lower than 30 were removed from the analysis.

### Impact prediction of regional changes

The preprocessing steps of the Basenji model^36^ were applied to transform BAM alignments to normalized BigWig coverage tracks (bam_cov.py). Next, train/test/validation sets of tracks were created using basenji_data.py utility and were saved as tensorflow records. In total, all the tracks were divided into 2366, 256 and 253 tracks in training, validation and test sets, respectively. The training code of Enformer^21^ was modified to accommodate our ATACseq data for rice and the network was trained for 10 batches of 150,000 steps.

To create the prediction tracks, the ID and coordinates of the DETs were extracted in a text file. In a python code, the kipoiseq module (https://github.com/kipoi/kipoiseq) was used to extract a sequence with length of 196,698 nucleotides centered on the transcription start site (TSS) of each DET. The testing code slid a window of 1000 nucleotides from the beginning to the end of the track in steps of one nucleotide. At every step, the contents of this sliding window were swapped with a sequence of random nucleotides. The in silico mutagenesis score (ISMS) was then calculated as the effect of mutation on the TSS of the target gene,

1 $${\text{ISMS }} = \, \left| {{\text{f}}\left( {{\text{modified}}} \right) \, - {\text{ f}}\left( {{\text{reference}}} \right)} \right|.$$

Here f is the predicted accessibility score of the model at the TSS. The significant peaks in this score are marked based on the threshold |z − score|< 3. To find the specific single nucleotide mutation that disrupts the expression of a target gene, the above mutagenesis approach was repeated in a single base resolution. In other words, every base was changed to all the other 3 possibilities and the maximum increase (minimum decrease) in the predicted probabilities was reported as the gain (loss) score for that base.

To find the transcription factor binding sites (TFBSs), the meme-motif of the TF bindings in rice was downloaded from PlantTFDB^37^. The sequences within prediction windows of each DEG was searched for occurrences of TFBSs using FIMO^38^ with high confidence (P < 10^−10^).

### Accessibility analysis

Using the BigWig tracks of the ATACseq data that were generated in the previous section, it can be confirmed whether the discovered DE-lncRNAs are from a region in the genome that is significantly accessible. This comparison, however, cannot be directly made, because the reported DE-lncRNAs are in the IRGSP-coordinates, whereas ATACseq tracks are generated in the ZS97 coordinate. As a result, the liftoff program^39^ was used to first lift the coordinates of DE-lncRNAs and all the other genes over to the ZS97 coordinates. Next, in a costume-written python code, the BigWig tracks from root were loaded using pyBigWig (https://github.com/deeptools/pyBigWig) and the lifted annotation was used to calculate the significance of the accessibility level of DE-lncRNAs.

### DE-lncRNAs acting prediction as miRNA target mimics

All identified DE-lncRNAs were submitted to the psRNATarget public web tool (https://plantgrn.noble.org/psRNATarget/analysis) to investigate whether DE-lncRNAs functioned as miRNA decoys. The identified interaction between DE-lncRNAs and osa-miRNAs was computed with less than four mismatches, maximum expectation = 5, and allowed maximum energy to unpair the target site (UPE) = 25^40^.

### Validation of DE-lncRNAs via quantitative real-time PCR (qRT-PCR) analysis

The cDNA library was synthesized using 1.5 μg of total RNA by iScriptTM cDNA synthesis kit (BioBasic) consistent with the manufacturer’s instructions before utilization in the qRT-PCR. The relative expression levels of DE-lncRNAs were analyzed by qRT-PCR using a LightCycler^®^ 96 Real-Time PCR System (Roche Life Science, Germany) and SYBR Premix BioFACT™ 2X (BIOFACT, South Korea) based on manufacturer’s instructions. To validate the results of our RNA-seq data, a sum of 7 DE-lncRNAs were randomly selected for qRT-PCR analysis. The specific primers for each DE-lncRNAs (Supplementary Table S1) were designed using Oligo 7.0 (ver. 5.0; National Bioscience Inc., Plymouth, USA). Rice ubiquitin gene (OS04G0628100) was used as reference gene. The relative expression of each transcript of DE-lncRNAs were computed by 2^−ΔΔCt^^41^. All methods were performed in accordance with relevant institutional (ABRII), national, and international guidelines and legislations.

## Results

### Identification of lncRNAs in the two rice genotypes

After eliminating low-quality reads, 111,183,429 and 105,132,619 clean reads in FL478 and IR29 rice genotypes remained for downstream analysis, respectively. Approximately, 40 million paired-end clean reads were mapped to the reference genome and assembled, as described in our previous study^22^. After mapping based on both indica and japonica genome references, the japonica genome reference was selected due to higher percentage mapping^23^. We performed a strict/novel pipeline to find high-confident lncRNA transcripts (Fig. 1). Firstly, 382,622 (96.15%) and 357,520 (95.59%) protein-encoding transcripts in FL478 and IR29 were respectively removed. Although more transcripts were mapped to the protein coding genes, 15,131 and 16,256 transcripts with class codes “u,” “o,” “i,” and “x,” which are most likely to be noncoding, were respectively selected in FL478 and IR29 cuffcompare output files (Supplementary Table S2). Then, we identified and removed some transcripts overlapping with other non-coding RNAs (including snRNAs, snoRNAs, and microRNAs etc.) in both genotypes (Supplementary Table S3). Subsequently, 256 (FL478) and 237 (IR29) transcripts with the length > 200 bp were nominated as potential long noncoding RNAs by CPC, and their highest and lowest lengths were detected approximately 3000 bp and 600 bp, respectively (Supplementary Table S4). The highest percent of potential lncRNAs was found in chromosome 1 in both genotypes (Supplementary Fig. S2). We also re-evaluated the selected non-coding transcripts through CPC with Pfam and SwissProt NR databases, resulting in excluding 39.84% and 36.70% of them in FL478 and IR29, respectively (Supplementary Table S4). Thus, 132 lncRNAs were found to be more frequent in FL478 than in IR29 (111 lncRNAs), of which 93 were sense lncRNAs, eight were anti-sense lncRNAs, 27 were lincRNAs, and four were intronic lncRNAs in FL478 (Supplementary Fig. S3). Similarly, sense lncRNAs (85) were the most frequent among the identified lncRNAs, while intronic lncRNAs (2) were the least frequent in IR29 (Supplementary Fig. S3).

### Identification of differentially expressed lncRNA

**Figure 2:**
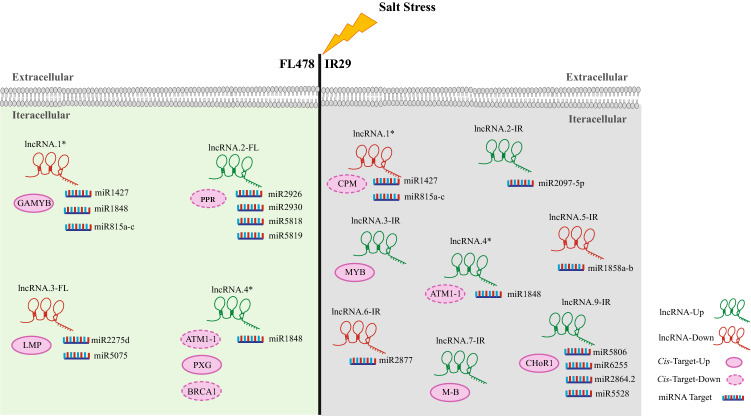
Schematic representation of lncRNAs and their targets including miRNA targets and *cis*-targets. (*LMP:* lipid metabolic process, *PPR:* pentatricopeptide repeat protein, *CMP:* component of plasma membrane, *M-B:* metal-binding).

Differentially expressed lncRNAs (DE-lncRNA) were identified by comparing samples collected at different situations (control and stress) in the root tissues to inspect the expression patterns of lncRNAs under salt stress conditions in the salt tolerant genotype (FL478) and its susceptible parent (IR29). Overall, only a few DE-lncRNAs were identified, with most of them being sense lncRNAs. A total of four DE-lncRNAs were detected in FL478, among which, two were up-regulated and two were down-regulated under salt conditions. Moreover, a total of nine DE-lncRNAs were found in IR29, including six up-regulated and three down-regulated DE-lncRNAs under salt stress (Table 1, Fig. 2). We further found two novel DE-lncRNAs in IR29 under salt stress (Table 1, Fig. 2). Two and seven out of the total DE-lncRNAs were exclusively expressed in FL478 and IR29, respectively (Table 1, Fig. 2). The comparative analysis of FL478 and IR29 lncRNAs in response to salt stress revealed that two DE-lncRNAs overlapped in both genotypes, although their lengths were shorter in FL478 than in IR29 (Table 1, Fig. 2).

**Table 1:** Details of the identified DE-lncRNA in FL478 and IR29 genotypes (the asterisk represents coincided DE-lncRNAs in both genotypes).

Genotype	Seq.ID	LncRNA.Name	Position			Length	log2 FC	Class Code
			Chr. No	Start	End			
FL478	TCONS_00038460	lncRNA.1*	2	9,106,571	9,107,483	912	− 1.84777	o
	TCONS_00088492	lncRNA.2-FL	6	21,764,438	21,765,266	828	1.33505	o
	TCONS_00110513	lncRNA.3-FL	9	19,868,030	19,868,740	710	− 1.84449	o
	TCONS_00069370	lncRNA.4*	4	25,515,690	25,516,406	716	1.3109	o
IR29	TCONS_00038953	lncRNA.1*	2	9,106,569	9,107,483	914	− 2.84421	o
	TCONS_00109665	lncRNA.2-IR	8	13,484,779	13,485,797	1018	2.43485	u
	TCONS_00014111	lncRNA.3-IR	1	23,750,606	23,751,636	1030	1.77214	o
	TCONS_00070354	lncRNA.4*	4	25,515,690	25,516,422	732	1.20362	o
	TCONS_00015930	lncRNA.5-IR	10	8,069,944	8,070,766	822	− 19.3908	o
	TCONS_00003216	lncRNA.6-IR	1	24,908,220	24,909,529	1309	− 1.70111	o
	TCONS_00073667	lncRNA.7-IR	4	24,983,145	24,983,890	745	2.11511	u
	TCONS_00028639	lncRNA.8-IR	11	2,932,659	2,933,342	683	1.21378	o
	TCONS_00076916	lncRNA.9-IR	5	26,093,812	26,095,238	1426	1.81551	o

### Chromatin accessibility of DE-lncRNAs

The logical necessary condition for the involvement of lncRNAs in the differential expression of target genes is the accessibility of their genomic region. All the 11 DE-lncRNAs were compared to the ATAC-seq tracks in the 6 tissues (Supplementary Fig. S4). These regions in the genome had elevated ATAC-seq levels in all tissues.

**Figure 3:**
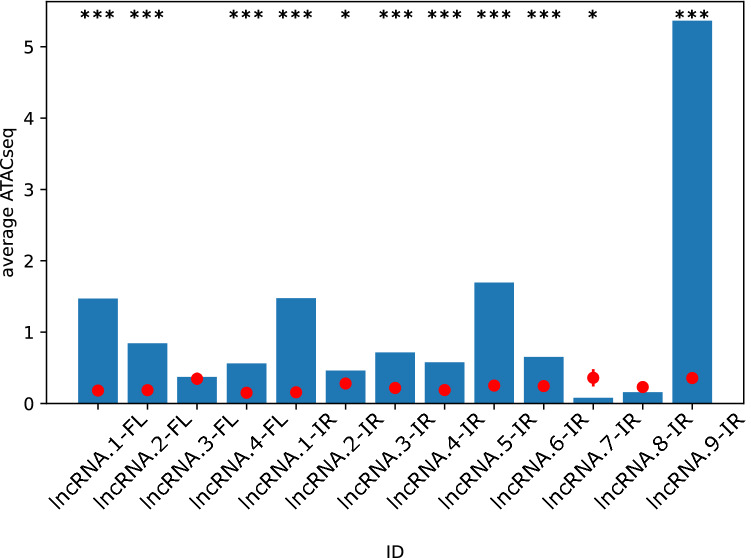
The significant accessibility of DE-lncRNAs in the genome. Average ATAC-seq signal in the 13 lncRNAs (bars) as compared to that of random locations with similar width in the genome (red dot). Error bars depict S.E.M. (*P < 0.05, ***P < 0.001).

To assess the genome-wide accessibility of DE-lncRNAs, the mean ATAC-seq signals across the length of each DE-lncRNAs were calculated (Fig. 3). In addition, to establish the significance of this result, for each DE-lncRNAs 50 regions of equal length with DE-lncRNAs were selected from random genomic positions and the average ATAC-seq signals were measured for them. The average signals for 3 out of 4 DE-lncRNAs in FL478 and 8 out of 9 DE-lncRNAs in IR29 were significantly higher than the random selection (Fig. 3).

### Functional prediction of salt stress‐responsive DE-lncRNAs involved in *trans*-regulation

**Figure 4:**
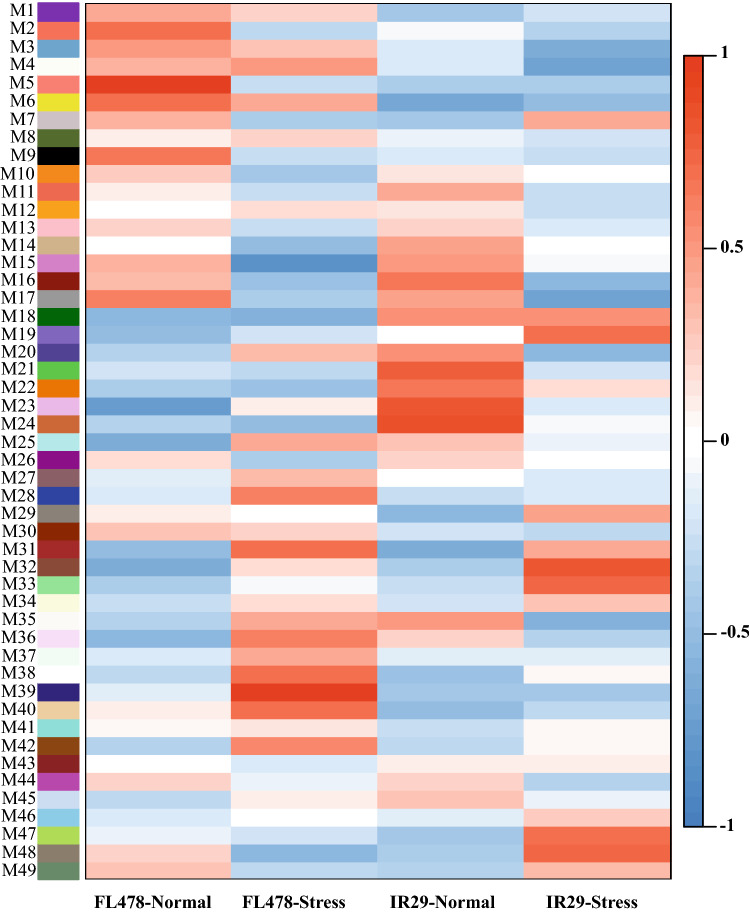
A visual representation of the 49 modules correlated with salt stress response. The heat-map scale reflects the correlation (r^2^) level among lncRNAs and mRNAs.

The potential functions of salt-responsive DE-lncRNAs were explored by detecting the *trans*-regulatory networks of DE-lncRNAs in each genotype. Overall, four (in FL478) and nine (in IR29) DE-lncRNAs, together with 4670 identified expressed transcripts (ETs) through RNAseq, were subjected to co-expression network analysis. WGCNA analyses identified 49 modules in the two genotypes. We found that lncRNA.2-FL was highly correlated with ETs in the transcriptional module M39 (r^2^ = 0.96, p < 1e − 04) in FL478 under salinity stress (Fig. 4). Intriguingly, the M39 module including lncRNA.2-FL and 173 ETs was significantly up-regulated in FL478 under salt stress whereas these genes (Supplementary Table S5) were down-regulated in IR29 (Fig. 4). Subsequently, the significantly enriched GO terms of lncRNA.2-FL co-expressed ETs (Supplementary Table S5) in the M39 module were identified using Fisher’s exact test (P < 0.05) revealing that they were mainly enriched for salinity stress-related categories. The GO terms "cell wall organization", "response to oxidative stress", "response to chemical stimulus" and "carbohydrate metabolic process" were enriched among biological processes (Supplementary Fig. S5). In the category of molecular functions, "hydrolase activity", "peroxidase activity", and "oxidoreductase activity" were indicated as dominant terms (Supplementary Fig. S5). In the cellular component, more transcripts were categorized in the "extracellular region" and "cytoplasm" (Supplementary Fig. S5). To get more insight into the function of lncRNA.2-FL targets, online KEGG automatic annotation server (KAAS) was used for target genes in the M39 module. The results indicated that 47 out of the 173 DETs were classified into 13 KEGG pathways related to "metabolism", "genetic information processing", and "signaling and cellular processes" (Supplementary Fig. S6). The term "enzyme" was predominantly enriched including glutathione S-transferase, phosphatase, dehydrogenase, and peroxidase under salt stress compared to normal conditions (Supplementary Fig. S6). In total, our results suggested that the M39 module may have a significant role in the salt tolerance of FL478. Notably, the M39 module was not significant in IR29 under normal and salinity treated conditions (Fig. 4).

It is worthy to note that 33 out of 173 ETs were significantly salt stress responsive (DETs, the Q-value cut-off ≤ 0.05 and − 1 ≥ log2 fold change ≤ 1 were set as the threshold for significantly differential expression) in FL478, of which 17 DETs were specifically expressed in FL478 (Supplementary Table S5).

### Functional prediction of salt stress‐responsive DE-lncRNAs involved in *cis*-regulation

To identify the putative functions of salinity-responsive DE-lncRNAs, the DE-lncRNAs were searched within 100 kb upstream and downstream for protein-encoding genes. Then, the DE-lncRNAs and their neighboring protein-coding genes were subjected to co-expression analysis. In total, seven lncRNA neighbors-protein-coding gene pairs were found to be involved in *cis*-acting regulation in FL478 (Supplementary Table S6, Fig. 2). Two protein-coding genes with 89 kb and 23 kb distance were found downstream of lncRNA.1; among them, LOC_Os02g16000 (log2 FC = 1.07, q-value = 0.01, and Cor = − 0.80) encoded a gene similar to GAMYB-binding protein (Supplementary Table S6, Fig. 2). Also, LOC_Os06g36910 (log2 FC = − 1.14, q-value = 0.05, and Cor = − 0.97), which is a pentatricopeptide repeat (PPR) protein, was located at 14 kb upstream of lncRNA.2-FL (Supplementary Table S6). Further, we found three protein-coding genes in the neighborhood of lncRNA.4, including LOC_Os04g43070 (log2 FC = − 2.42, q-value = 0.001, and Cor = − 0.94) encoding AMT1-1 located 15 kb downstream of lncRNA.4 whose function is response to abscisic acid, LOC_Os04g43300 (log2 FC = − 1.14, q-value = 0.01, and Cor = − 0.87) encoding *BRCA1* located 95 kb upstream of lncRNA.4 whose function is positive regulation of transcription, and LOC_Os04g43200 (log2 FC = 5.55, q-value = 0.001, and Cor = 0.90) encoding peroxygenase (*PXG*) located 47 kb upstream of lncRNA.4 whose function is calcium-binding peroxygenase (Supplementary Table S6, Fig. 2).

Likewise, we identified five lncRNA–mRNA pairs involved in *cis*-acting regulation in IR29. LOC_Os02g16030 (log2 FC = − 2.84421, q-value = 0.0012, and Cor = 0.92673) encoding a plasma membrane component, located three bp upstream of lncRNA.1 (Supplementary Table S6). LOC_Os01g41900.1 (log2 FC = 1.38, q-value = 0.001, and Cor = 0.87) encoding the MYB transcription factor was located 8 kb upstream of lncRNA.3-IR (Supplementary Table S6). LOC_Os04g42020 (log2 FC = 1.01357, q-value = 0.003, and Cor = − 0.9186) whose function is zinc binding was spaced 93163 bp downstream of lncRNA.7-IR (Supplementary Table S6). Also, LOC_Os05g45030 (log2 FC = 1.30, q-value = 0.011, and Cor = 0.56) encoding *calcium homeostasis regulator* (*CHoR*) was located 81 kb upstream of lncRNA.9-IR (Supplementary Table S6, Fig. 2). Calcium, known as an essential plant element, is related to adaptive responses against environmental stresses^42^. These results showed that DE-lncRNAs might *cis*-regulate their neighboring protein-encoding genes’ expression in response to salt stress in both genotypes.

### Impact assessment of possible mutations in DE-lncRNAs on *cis*-regulatory target genes

To find a causal relationship between DE-lncRNAs and their *cis*-regulatory target genes, we used a recently developed DNN^21^ to predict the change in the expression levels of the genes as a result of introducing mutations in DE-lncRNAs. We retrained this Enformer network with the ATAC-seq tracks of six tissues in rice^33^. Further, our trained Enformer model was used to predict the changes in accessibility of the genetic regions as a result of introduction of mutations in the DE-lncRNAs.

**Figure 5:**
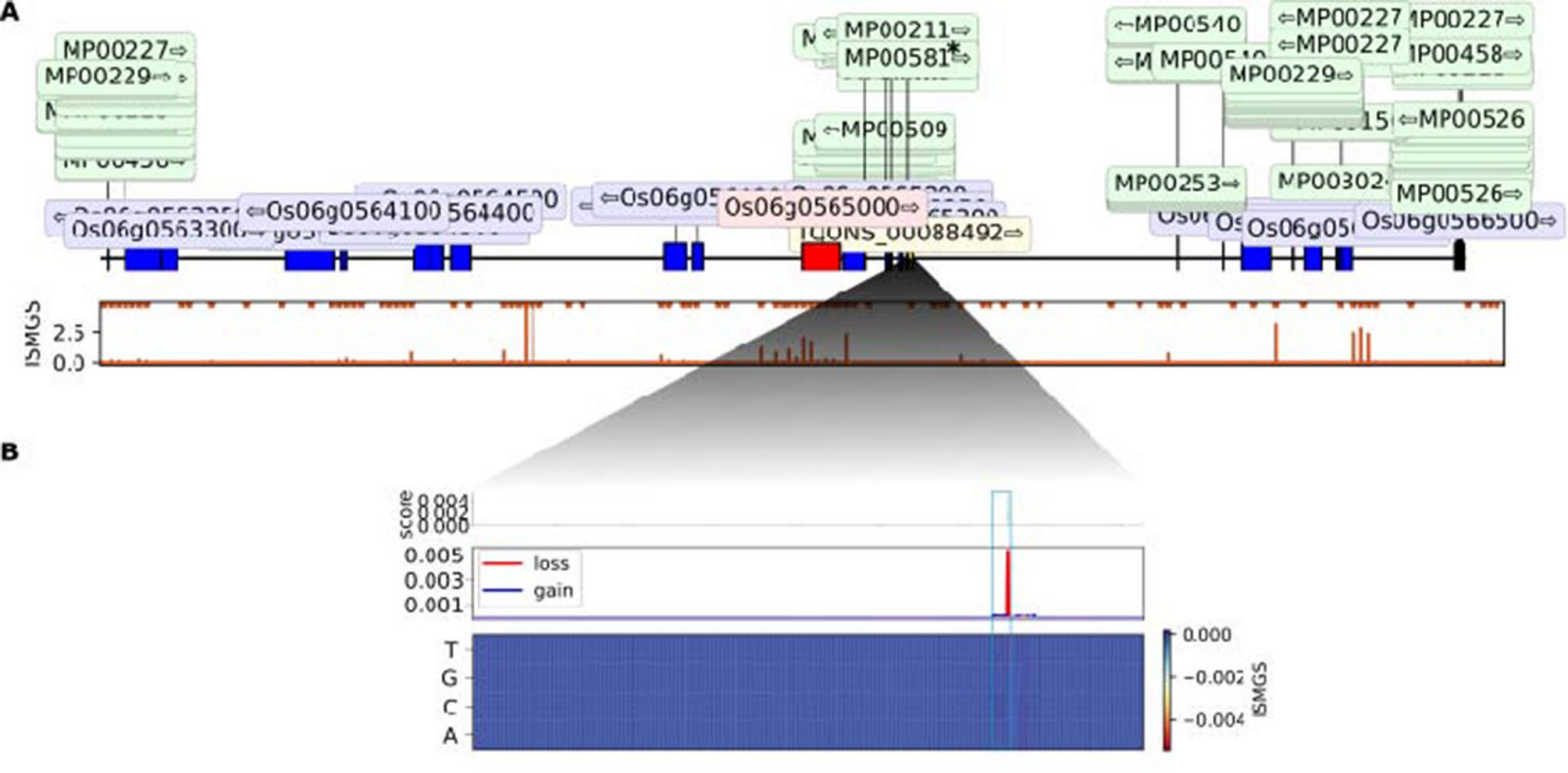
In silico mutagenesis of lncRNA.2-FL. (**A**) The top panel shows a 200 kb neighboring window around *cis*-regulatory target gene “Os06g0565000” (red). All the other genes are marked in blue, transcription factors (TFs) in green and lncRNA.2-FL in yellow. The predicted mutagenesis score and its significant peaks is shown in the panel below. (**B**) This panel display mutagenesis of every base within lncRNA.2-FL to other possibilities and their effect on the TSS of Os06g0565000. The location of TF MP00581 is marked with a blue box.

The model predicts that only a window of lncRNA.2-FL had a significant prediction peak in ISMS that can affect the TSS of Os06g0565000 (LOC_Os06g36910) (Fig. 5). LOC_Os06g36910 is a PPR-coding gene. PPR proteins are as RNA-binding proteins, which participate in posttranscriptional processes such as RNA editing, splicing, stability, cleavage, degradation, and translation^43^.

### Mining the common TFBS in the *cis* and *trans*-regulatory target genes

To get insight into the regulatory mechanisms by which lncRNA.2-FL influence its target genes, point mutation analysis was employed to find TFBSs in the neighborhood of the *cis* and *trans*-regulatory target genes of lncRNA.2-FL that significantly affect the TSS of the genes. We found five TFBSs in lncRNA.2-FL that coincided with ISMS prediction peaks to affect the expression of its *cis*-regulatory target gene (Os06g0565000 (LOC_Os06g36910)) (Fig. 5). These binding sites belong to LBD and ERF TF families (Table 2).

**Table 2:** Summary of TFBSs located near the *cis*-regulatory target gene of lncRNA.2-FL.

TFBS		Up/down	Distance	TF.ID	TF family
Start	End				
21,764,521	21,764,541	Upstream	115	LOC_Os01g07480	LBD (lateral organ boundaries)
21,764,524	21,764,544	Upstream	111	LOC_Os01g07480	LBD (lateral organ boundaries)
21,764,522	21,764,542	Upstream	101	LOC_Os01g66590	LBD (lateral organ boundaries)
21,764,522	21,764,541	Downstream	102	LOC_Os02g42585	ERF (ethylene-responsive factors)
21,764,523	21,764,542	Upstream	101	LOC_Os11g13840	ERF (ethylene-responsive factors)

**Figure 6:**
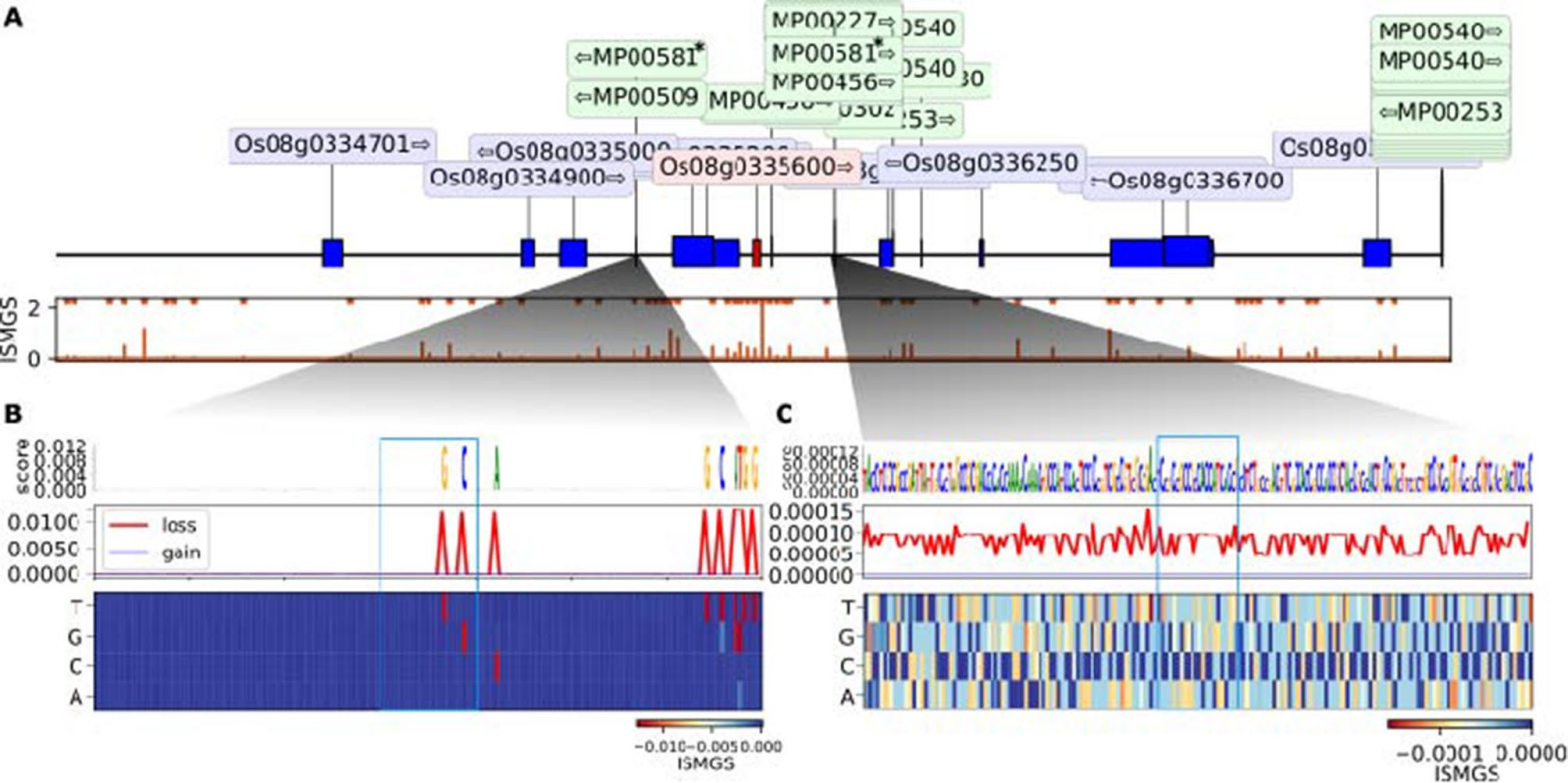
In silico mutagenesis of *OSAIR12*. (**A**) (top panel) A 200 kb neighboring window around *trans*-regulatory target gene “*OSAIR12*” (red). All the other genes are marked in blue and transcription factors (TFs) in green. The predicted mutagenesis score and its significant peaks is shown in the panel below. (**B**,**C**) Mutagenesis of every base within two *cis* regions to other possibilities and their effect on the TSS of *OSAIR12*. The locations of TF MP00581 are marked with blue boxes.

These five TFBSs were also found on the ISMS prediction peaks of the 12 out of 17 specific *trans*-regulatory target genes of FL478 (Table 3, Supplementary Table S5). Notably, LOC Os01g07480 encoding an LBD TF was spotted to affect “*OSAIR12*” and “*OSAPP1*” *trans*-regulatory target genes (Fig. 6b,c; Table 3). Similar mutagenesis analysis around these genes further support the causal link of this TF in the expression of LOC Os01g07480 (Fig. 6).

**Table 3:** The specific *trans*-regulatory target genes of FL478 with common TFBS in *cis*-regulatory target gene of lncRNA.2-FL.

No	Trans-regulatory target genes	Locus	log2 FC	q_value	Description
1	OS05G0541000	5:26,858,392–26,860,247	1.03185	0.009655	–
2	OS12G0137700	12:1,830,783–1,832,039	1.07775	0.029418	Sulfotransferase activity
3	OS07G0469200	7:16,748,940–16,749,713	1.08593	0.028187	–
4	OS01G0198900	1:5,373,582–5,374,794	1.43494	0.001754	Lachrymatory factor synthase
5	OS09G0425400	9:15,426,338–15,426,865	1.47524	0.001754	Keratin, type I cytoskeletal 9
6	OS04G0483500	4:24,175,619–24,178,976	1.57775	0.004489	Oxidoreductase/dehydrogenase
7	OS07G0599700	7:24,477,362–24,478,154	1.85873	0.001754	IgA FC receptor precursor, Signal
8	OS03G0167000	3:3,626,392–3,628,635	2.12414	0.001754	LTPL82
9	OS08G0335600	8:15,008,763–15,009,754	2.67403	0.001754	*OSAIR12*, Signal
10	Os08g0136300	8:2,054,222–2,055,830	3.38831	0.011481	–
11	OS07G0142600	7:2,184,248–2,184,998	3.84603	0.006691	*OSAPP1*, Signal
12	OS10G0173000	10:4,994,498–4,995,160	4.65991	0.005628	Integral component of membrane

### Salt responsive lncRNAs as potential targets of rice miRNAs

The crosstalk of DE-lncRNAs and miRNAs was inspected through exploring the DE-lncRNAs regarded as the target pattern of known miRNAs in *Oryza sativa*. All the DE-lncRNAs in FL478 were identified to act as target patterns of 13 known osa-miRNAs, including osa-miR1427, osa-miR1848, osa-miR2275d, and osa-miR2926 (Supplementary Table S7, Fig. 2). Also, six out of nine DE-lncRNAs in IR29 were recognized as target patterns of 16 known miRNAs, including osa-miR2877, osa-miR1858, and osa-miR1427 (Supplementary Table S7, Fig. 2). The comparative analysis of these miRNAs in the two contrasting genotypes revealed that five and eight miRNAs were exclusively found in FL478 and IR29, respectively (Supplementary Table S7, Supplementary Fig. S7, Fig. 2) whereas eight miRNAs were commonly identified in both genotypes. Among the miRNAs that specifically identified in FL478, the target genes of two miRNAs, including osa-miR2926 and osa-miR2930, were predicted to be associated with lncRNA.2-FL (Supplementary Table S7, Fig. 2). As shown in Supplementary Table S7, multiple interactions were identified between lncRNA.2-FL with osa-miR2926 and osa-miR2930 with many mRNAs. Among them, genes controlling potassium and chloride channels were found, which may have potential roles in salt stress tolerance (Supplementary Table S7). Also, gene encoding glutathione S-transferase was previously predicted as target of osa-miR2275d related to lncRNA.3-FL (down-regulated) (Supplementary Table S7, Fig. 2)^44^. The interactions between common DE-LncRNAs and miRNAs predicted 5 and 3 known miRNAs target related to lncRNA.1 and lncRNA.4, respectively (Supplementary Table S7). GO analysis of miRNAs target genes associated with lncRNA.1 which was down-regulated in both genotypes suggested that the genes were significantly enriched in catalytic activity term (Supplementary Table S8). Also, many miRNAs target genes were predicted for lncRNA.4; GO analysis of these target genes suggested that heme binding term was most significant term as a probabilistic function of lncRNA.4 (p-value = 0.00096 and FDR = 0.039) (Supplementary Table S8).

### Reliability assessment of RNA-seq based inferences through qRT-PCR

**Figure 7:**
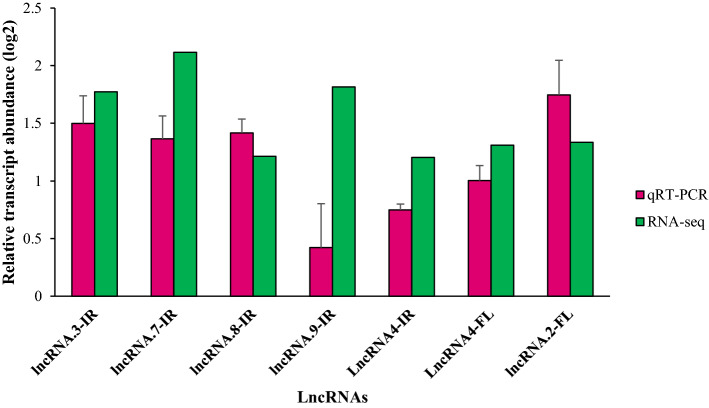
Validation of DE-lncRNAs using qRT-PCR in root tissues of FL478 (tolerant genotype) and IR29 (sensitive genotype). Bar graphs represent the relative transcript abundance of DE-lncRNAs based on three biological replicates, the standard deviation of the relative expression levels (n = 3) is indicated by error bars.

To verify analysis of RNA-seq data, 7 DE-lncRNAs were randomly nominated for qRT-PCR. Overall, the qRT-PCR results confirmed the outcome of RNA-seq analysis (Fig. 7). The qRT-PCR data revealed that 5 DE-lncRNAs from IR29 were up-regulated in 24 h after the onset of salt stress, among which lncRNA.3-IR, lncRNA.7-IR, lncRNA.8-IR and lncRNA.4 were highly consistent with the RNA sequencing results (Fig. 7). Similarly, in FL478, the expression levels of two DE-lncRNAs including lncRNA.2-FL and lncRNA.4 were confirmed by qRT-PCR (Fig. 7).

## Discussion

Rice is a significant crop, accounting for food security, and a model plant. Nevertheless, salt stress leads to a significant yield loss in rice. LncRNAs have been raised as important regulatory factors in response to salt stress, though rice lncRNAs have been poorly examined. In the present study, lncRNAs were identified at the whole transcriptome level in FL478 as a source of salt tolerance at the seedling stage in rice compared to its susceptible parent (IR29). Totally, 11 DE-lncRNAs (four DE-lncRNAs in FL478 and nine DE-lncRNAs in IR29) were identified, which may play a crucial role in rice’s response to salt stress. Consistent with the previous reports^22,45^, susceptible plants’ response to stress is more prevalent, comprising a higher number of DETs/DE-lncRNAs, due to less efficient mechanisms to cope with the stress conditions, leading to greater and faster confrontation of cells to the tension. However, tolerant genotype response is more specific and proficient.

### The DE-lncRNAs may play some roles in salt responses through their *cis*-targets

Previous studies reported that lncRNAs could *cis*-regulate nearby regulatory elements such as promoters and enhancers^17,46^. In this study, seven and five co-expressed protein-coding genes were identified in adjacent to DE-lncRNAs in Fl478 and IR29 genotypes, respectively, while only one of them (*AMT1-1*) related to lncRNA.4 was in common (Supplementary Table S6). A gene encoding gibberellin-dependent alpha-amylase expression (*GAMYB*) was found nearby ncRNA.1 (Supplementary Table S6), while *GAMYB* was annotated as a salt responsive gene^47^. Remarkably, a gene coding pentatricopeptide repeat protein (PPR) was found as *cis*-target of lncRNA.2-FL (Supplementary Table S6). The PPR family is one of the largest plant protein families, which contains 477 proteins in rice (*Oryza sativa* L.). PPR proteins are RNA-binding proteins, which participate in multiple posttranscriptional modification such as RNA editing, splicing, stability, cleavage, degradation, and translation. It has been reported that almost all PPR proteins are located and function in chloroplasts or mitochondria, of which some PPR proteins were reported to be involved in RNA splicing or editing of genes under abiotic stress including salt or drought stress responses in rice^43,48^. *AMT1-1* was co-expressed with and located nearby lncRNA.4 in both Fl478 and IR29. It is reported that *AMT1-1* can regulate rice growth and NH4^+^ uptake through brassinosteroid (BR) signaling pathway^49^. Also, two genes encoding *PXG* and *BRCA1* were found nearby of lncRNA.4 in FL478 (Supplementary Table S6). It has reported that Ca^2+^-dependent peroxygenase is induced by drought, high salinity and ABA in Arabidopsis, which is actively involved in a wide range of physiological functions^50^. Moreover, *BRCA1* involved in positive regulation of transcription by RNA polymerase II, multiple cellular processes such as DNA repair, chromosome segregation and chromatin remodeling (Supplementary Table S6)^51^. These results suggest that the DE-lncRNAs play some roles in salt responses by regulating these neighboring genes with functions such as ROS scavenging, transcription and post-transcriptional regulation, protein folding and transport.

### The miRNAs mediating some functions of the DE-LncRNAs

**Figure 8:**
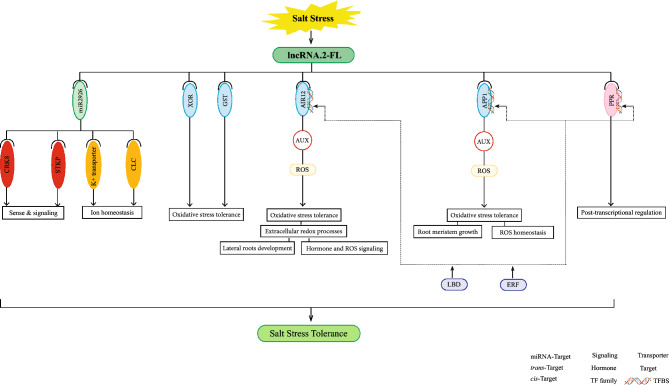
The hypothetical model for regulatory network through lncRNA.2-FL in FL478 under salt stress.

The interactions between lncRNAs and miRNAs are critical for various biological events. Thus, exploring these interactions helps us understand lncRNAs’ functions^52^. It is revealed that LncRNAs may act through miRNAs for transcriptional, post-transcriptional, and epigenetic gene regulation. In this study, all four DE-lncRNAs in FL478 and six out of nine DE-lncRNAs in IR29 were predicted to act as target patterns of 13 and 16 known osa-miRNAs, respectively (Supplementary Table S7). Among the miRNAs predicted to be exclusively associated with DE-lncRNAs in FL478, osa-miR2926 related to lncRNA.2-FL is most probably supposed to be involved in salt stress tolerance (Fig. 8; Supplementary Table S7). Osa-miR2926 is predicted to target many salt stress responsive genes, such as genes encoding chloride channel protein (CLC), potassium transporter and some genes involved in sensing and signaling such as cysteine-rich receptor-like protein kinase 8 precursor (CRK8) and serine/threonine protein kinase (STKP) (Supplementary Table S7). Previous studies showed that receptor-like kinases are significant signaling components, which regulate multiple cellular processes^53,54^. It is reported that miR2926 plays a major role in abiotic stress conditions based on the functional annotation of its target genes^55^. Previous reports showed that *OsCLC1* was up-regulated under salt stress in roots of Pokkali (rice salt tolerant genotype), while it was down-regulated in IR29 (salt sensitive genotype). It is suggested that *OsCLC1* regulates anion and cation homeostasis in Pokkali^56^. Enhancing K^+^ uptake is critical for the survival of glycophytes under toxic accumulation of Na^+^ in salt environment. Thus, the potassium transporter significantly contributes to the salt stress tolerance of these plants^57^. We therefore argue that the interaction between lncRNA.2 and osa-miR2926 might be related to the expression of genes involved in NA^+^/K^+^ homeostasis and signaling in FL478 under salt stress, although our current knowledge is still limited (Supplementary Table S7).

### LncRNA.2-FL as a candidate regulator of salt tolerance in FL478

It is reported that lncRNAs can regulate the expression level of unlinked genes in the genome through interaction with promoters and enhancers, or proteins that bind to these sites, affecting chromatin states and RNA polymerase activity^58^. Based on the previous reports, the mechanism of lncRNAs in stress response could be predicted by functions of their targets^59,60^. According to co-expression network analyses in the current study, it was revealed that the lncRNA.2-FL expression was highly correlated with the M39 transcriptional module under salinity stress, of which 17 DETs were highly salt responsive, exclusively in FL478 (Fig. 4, Supplementary Table S5). Based on the GO and KEGG pathway analysis results, functions of the genes located in the M39 module were mainly associated with salt stress tolerance, such as oxidative stress-related responsive genes, aquaporins, and cell wall-related genes. Among the DETs of M39 module, some involved genes in ROS signaling were found such as *glutathione S-transferase* (*GST*)*, OSAIR12*, *OSAPP1* and *oxidoreductase* (*XOR*) (Supplementary Table S5). Glutathione S-transferases play important roles in oxidative stress tolerance and cellular detoxification^61^. *AUXIN INDUCED IN ROOTS* (*AIR12*) encoding glycosylphosphatidylinositol tail anchored protein has been associated with extracellular redox processes and influences primary and lateral root development through auxins as a critical regulator^62,63^. Further, *AIR12* is known as an extracellular constituent connecting both hormone and ROS signaling in plants under abiotic stress^62^. In addition, p-Loop NTPase encoded by mitochondria localized *APP1* regulates the maintenance of the root apical meristem through controlling local ROS homeostasis^64,65^. Moreover, in the root apical meristem, ROS and auxin signaling are contrastingly regulated to equilibrate root meristem growth^64^. Given the significant correlation between lncRNA.2-FL and these genes in M39 module, lncRNA.2-FL may act at root development through controlling ROS homeostasis and auxin signaling.

### Confirming functional importance of lncRNA.2-FL using a DNN-based model

Discovering targets of lncRNAs and understanding their mechanisms through experimental methods are costly and time-consuming. Yet, few in silico models for prediction of functional mechanisms of plant lncRNAs are proposed, while a reliable and powerful one is highly required. In this study, we used a recently developed deep neural network, Enformer, to predict the functional importance of lncRNAs genomic regions on the expression level of target genes. We trained this model using the ATAC-seq data from six rice tissues.

As lncRNAs are known to control the expression and function of their nearby genes^14^, we used our trained model to investigate their role on the expression of their *cis*-target genes. Our model predicted that introducing random mutations in lncRNA.2-FL could significantly affect the accessibility level of the transcription start site (TSS) of its *cis*-target gene (encoding PPR).

To further understand the regulatory mechanism by which lncRNA.2-FL affects its *cis*-target gene, we searched for transcription factor binding sites (TFBSs) within its genomic region. We found binding sites for the transcription factors belonging to the Lateral organ Boundaries Domain (LBD) and Ethylene Responsive Factor (ERF) families in the neighborhood of PPR-coding gene (*cis*-target of lncRNA.2-FL). Single site mutagenesis analysis by our model predicted that mutations within these TFBSs would have significant negative effect on the TSS of the *cis*-target (the peak in loss score, Fig. 5).

Interestingly, we found the same TFBSs near the *trans*-regulatory target genes of lncRNA.2-FL, which significantly affect the TSS of these genes. Single-site mutagenesis analysis of these TFBSs also confirmed their crucial influence on the trans-regulatory targets of lncRNA.2-FL such as genes coding *OSAIR12* and *OSAPP1*.

LBD family proteins regulate a large number of developmental and metabolic processes such as lateral root formation. In addition, a subset of LBD members play vital function in Aux- triggered root development. The Aux–LBD module through Aux/IAA-ARF pathway directly regulates lateral root organogenesis in Aux signaling^66^. Some genes involved in this module coding IAAs and ARFs (for example *AUX* and *PIN*) were previously found to be differentially expressed in FL478 in our previous study^22^. ERF transcription factors have an essential role in setout of responses to abiotic stresses in rice. Synthesis of ethylene as plant stress hormone is induced by diverse environmental stresses, like salinity. It is reported that ethylene raises salinity tolerance through increasing ROS scavenging or by gathering of ROS in root-vasculature specific leading to reduction of ROS accumulation and enhancing Na/K homeostasis^67^.

This evidence suggested that all these might be related to signaling pathways of halotropic movements. Halotropism is a newly discovered salt avoidance tropism, which allows plant to runaway from salinity by bending. Halotropism can assist plant to remodel root system architecture (RSA) to survive under salt condition^68^.

## Conclusions

Based on the transcriptome analysis of two rice contrasting genotypes, some DE-lncRNAs were found that are supposed to be involved in rice’s response to salt stress. Among them LncRNA.2-FL might play some roles in salt stress tolerance of FL478 by regulating sense and signaling, ion homeostasis and oxidative stress tolerance (Fig. 8). LncRNA.2-FL and its regulatory targets might have a role in lateral root formation through redistribution of auxin in the root to avoid a high salt concentration. Further, their functions in ROS and auxin signaling boost well-timed sense of tension in FL478 under salinity. All these lead to proficient responses of FL478 to salt stress compared to its susceptible parent (IR29).

## Supplementary Information

Supplementary Figures.

Supplementary Table S1.

Supplementary Table S2.

Supplementary Table S3.

Supplementary Table S4.

Supplementary Table S5.

Supplementary Table S6.

Supplementary Table S7.

Supplementary Table S8.

## Data Availability

Accession codes: All primary sequence read data has been deposited in NCBI database under BioProject ID: PRJNA493951 and PRJNA493923.
