# Supplementary material for: Long non-coding RNAs as the regulatory hubs in rice response to salt stress
Source: Sci Rep. 2022 Dec 15;12:21696. doi: 10.1038/s41598-022-26133-x (PMC9755261; doi:10.1038/s41598-022-26133-x)
Supplement: Supplementary file 1 — Supplementary Figures. [file 41598_2022_26133_MOESM1_ESM.pdf]

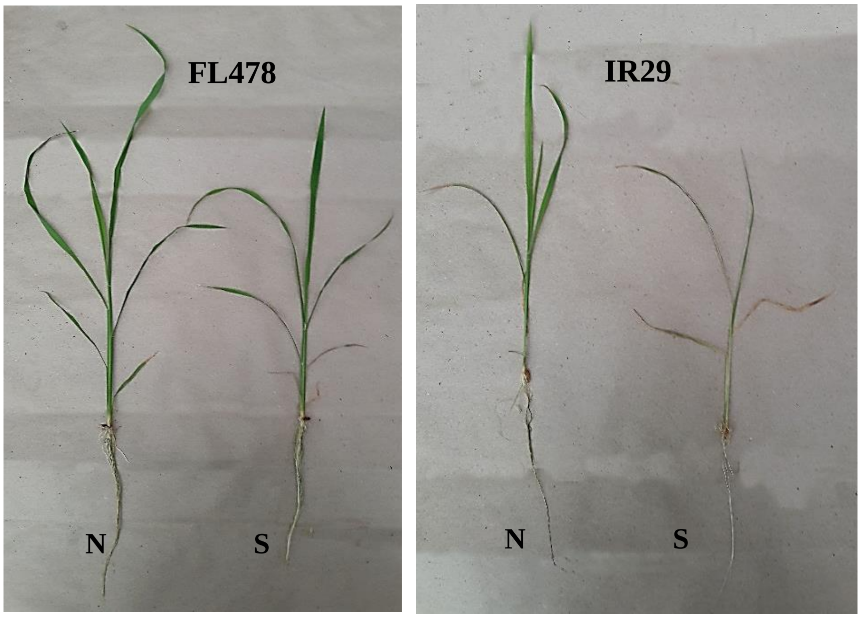

Fig. S1. Phenotypic response of FL478 and IR29 at normal conditions (N) and 1 week after the onset of salinity stress (S).

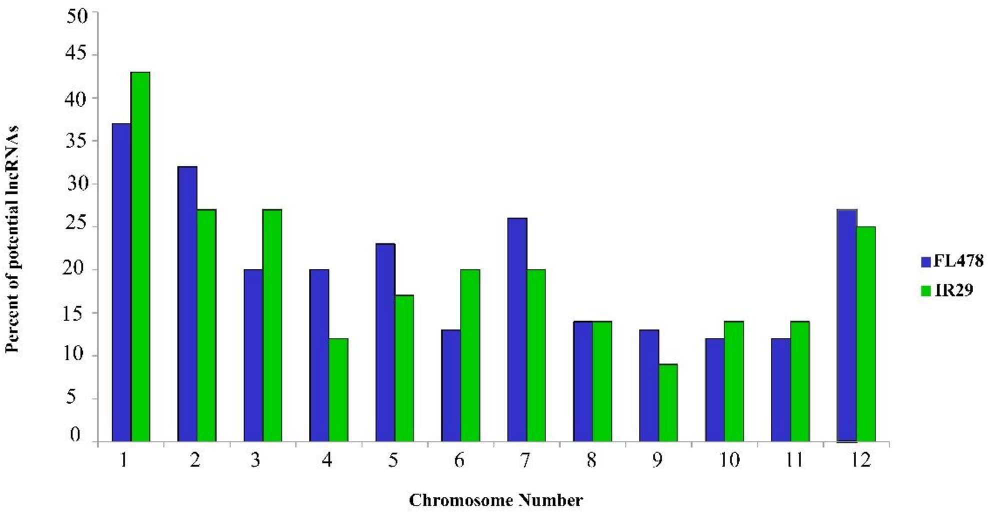

Fig. S2. Distribution of the expressed lncRNAs related to salt responses in each chromosome in the rice genotypes' roots.

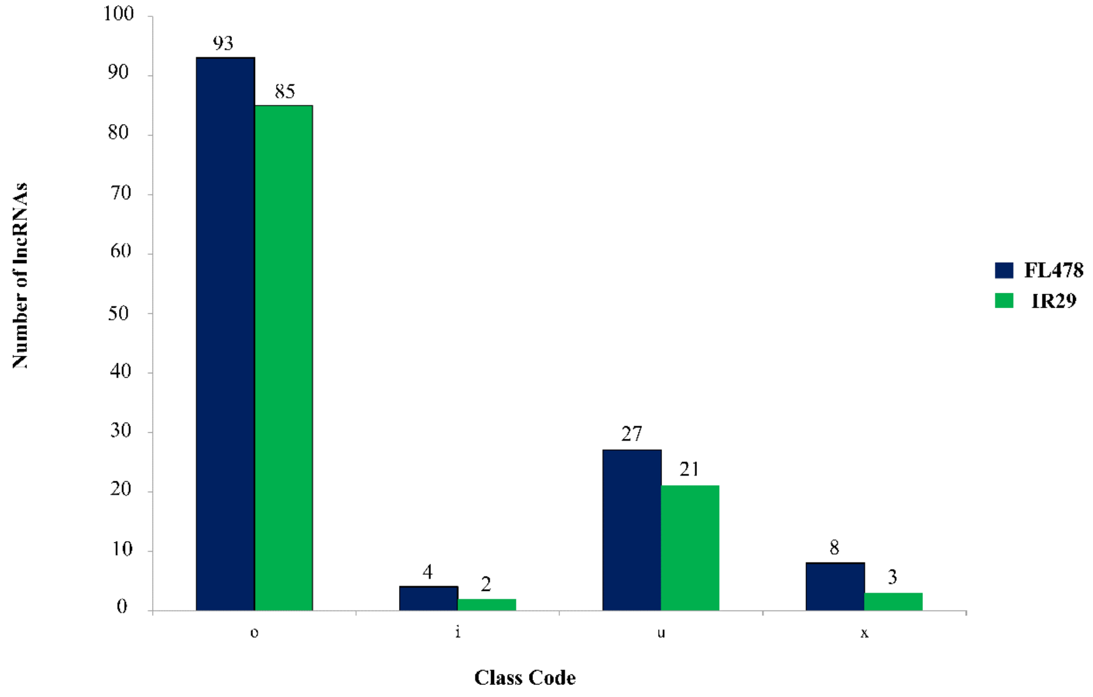

Fig. S3. The lncRNAs classification according to their position and orientation in the rice genome. Class code; ‘o’: lncRNAs that had exonic overlap with a known transcript, ‘i’: Intronic lncRNAs that are not sharing any sequences with exons, ‘u’: intergenic lncRNAs, ‘x’: exonic lncRNAs present on the opposite strand (antisense).

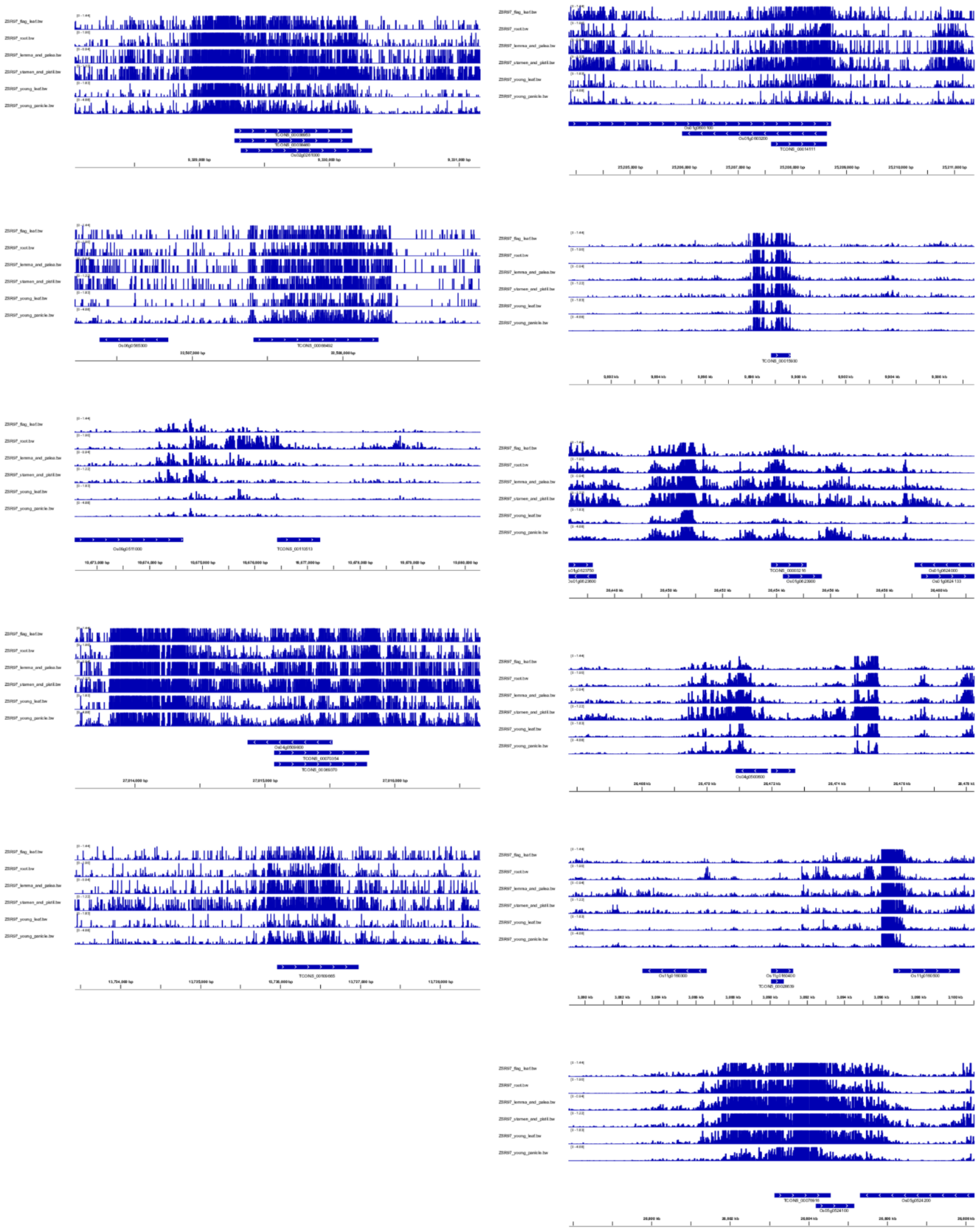

Fig. S4. ATAC-seq levels on the genomic regions of all the 11 DE-lncRNAs in 6 tissues.

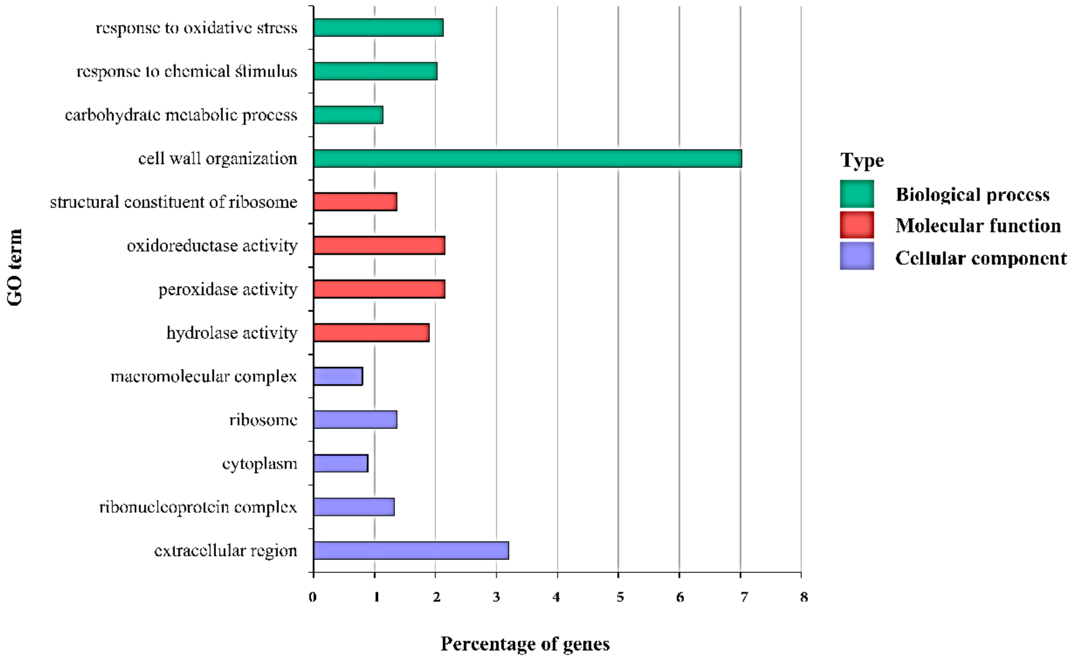

Fig. S5. The gene ontology (GO) enrichment analysis for target genes in the M39 module.

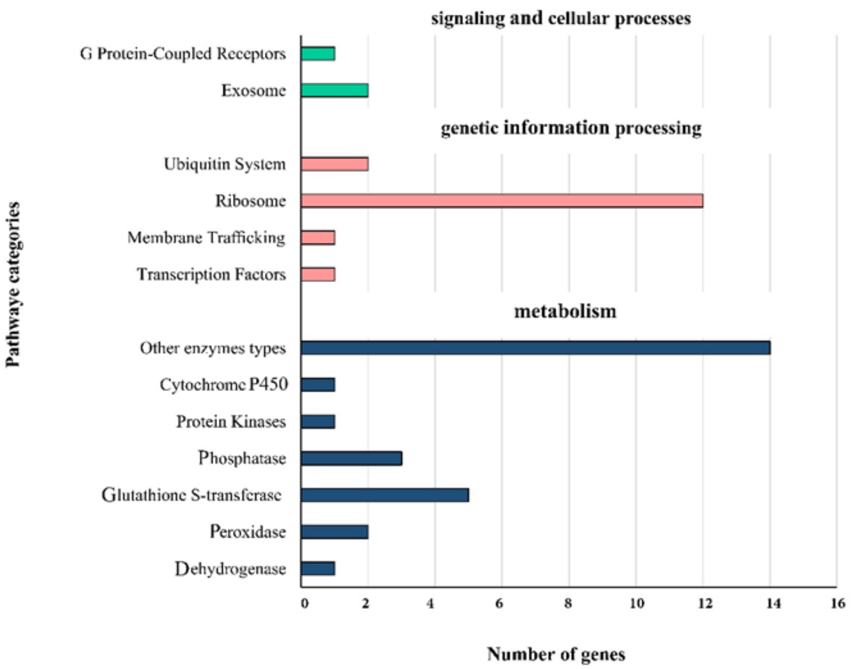

Fig. S6. Distribution of the KEGG pathways for target genes in the M39 module into three main categories.

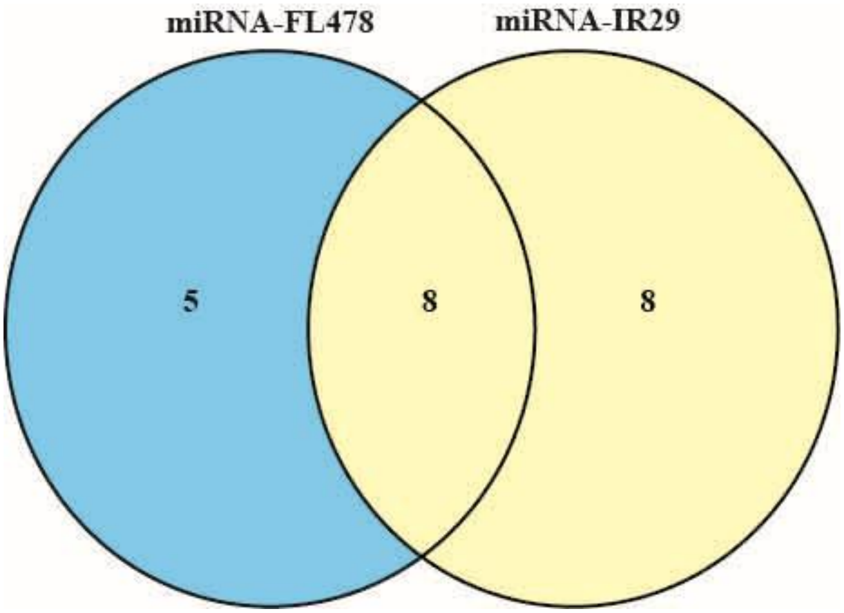

Fig. S7. A Venn diagram showing potential target miRNAs in the studied contrasting genotypes.
