# Supplementary material for: Long non-coding RNAs as the regulatory hubs in rice response to salt stress
Source: Sci Rep. 2022 Dec 15;12:21696. doi: 10.1038/s41598-022-26133-x (PMC9755261; doi:10.1038/s41598-022-26133-x)
Supplement: Supplementary file 9 — Supplementary Table S8. [file 41598_2022_26133_MOESM9_ESM.docx]

| **GO term** | **Ontology** | **Description** | **Number in input list** | **Number in BG/Ref** | **p-value** | **FDR** |
| --- | --- | --- | --- | --- | --- | --- |
| GO:0003824 | F | catalytic activity | 66 | 12146 | 0.00011 | 0.02 |

**Table S8.** Go analysis of miRNAs target genes related to a) lncRNA.1, and b) lncRNA.4.

**a:**

**b:**

| **GO term** | **Ontology** | **Description** | **Number in input list** | **Number in BG/Ref** | **p-value** | **FDR** |
| --- | --- | --- | --- | --- | --- | --- |
| GO:0004497 | F | monooxygenase activity | 7 | 467 | 0.0004 | 0.039 |
| GO:0020037 | F | heme binding | 8 | 707 | 0.00096 | 0.039 |
| GO:0003824 | F | catalytic activity | 51 | 12146 | 0.0011 | 0.039 |
| GO:0046906 | F | tetrapyrrole binding | 8 | 714 | 0.001 | 0.039 |
